# The Duodenum-Centered Neurohormonal Hypothesis of Type 2 Diabetes: A Mechanistic Review and Therapeutic Perspective

**DOI:** 10.3390/cimb47080657

**Published:** 2025-08-14

**Authors:** Athena N. Kapralou, Christos Yapijakis, George P. Chrousos

**Affiliations:** 1 General Surgery Department, Euroclinic Hospital, 115 21 Athens, Greece; 2 Unit of Orofacial Genetics, 1st Department of Pediatrics, School of Medicine, National Kapodistrian University of Athens, 115 27 Athens, Greece; cyapi@med.uoa.gr; 3 Endocrine Unit, UNESCO Chair on Adolescent Health Care, University Research Institute of Maternal and Child Health and Precision Medicine, National and Kapodistrian University of Athens, 115 27 Athens, Greece; chrousge@med.uoa.gr; 4 Hellenic Pasteur Institute, 115 21 Athens, Greece

**Keywords:** digestion, high-fat diet, insulin resistance, type 2 diabetes mellitus, biliopancreatic secretion, vagus nerve, pancreatic enzymes, bile acid, cholecystokinin, secretin, vagotomy, proximal intestinal bypass, gastric bypass

## Abstract

Type 2 diabetes mellitus (T2DM) is a multifactorial disorder defined by insulin resistance, β-cell dysfunction, and chronic hyperglycemia. Although peripheral mechanisms have been extensively studied, increasing evidence implicates the gastrointestinal tract in disease onset. Insights from bariatric surgery, gut hormone signaling, and incretin-based therapies suggest that the gut contributes actively beyond nutrient absorption. Yet, a cohesive framework integrating these observations remains absent, leaving a critical gap in our understanding of T2DM’s upstream pathophysiology. This work builds upon the anti-incretin theory, which posits that nutrient-stimulated neurohormonal signals—termed “anti-incretins”—arise from the proximal intestine to counteract incretin effects and regulate glycemic homeostasis. The excess of anti-incretin signals, perhaps stimulated by macronutrient composition or chemical additives of modern diets, disrupts this balance and may cause insulin resistance and β-cell depletion, leading to T2D. We hypothesize that the neuroendocrine signals produced by cholecystokinin (CCK)-I and secretin-S cells, both located in the proximal intestine, function as endogenous anti-incretins. In this context, we hypothesize a novel model centered on the chronic overstimulation of I and S cells by high-fat, high glycemic index modern diets. This drives what we term “amplified digestion”—a state marked by heightened vagal and hormonal stimulation of biliary and pancreatic secretions, increased enzymatic and bile acid activity, and alterations in bile acid composition. This condition leads to an extended breakdown of carbohydrates, lipids, and proteins into absorbable units, thereby promoting excessive nutrient absorption and ultimately contributing to insulin resistance and progressive β-cell failure. Multiple lines of clinical, surgical, and experimental evidence converge to support our model, rooted in the physiology of digestion and absorption. Western dietary patterns appear to induce an over-digestive adaptation—marked by excessive vagal and hormonal stimulation of biliary and pancreatic secretion—which amplifies digestive signaling. This heightened state correlates with increased nutrient absorption, insulin resistance, and β-cell dysfunction. Interventions that disrupt this maladaptive signaling—such as truncal vagotomy combined with duodenal bypass—may offer novel, physiology-based strategies for T2DM treatment. This hypothesis outlines a potential upstream contributor to insulin resistance and T2DM, grounded in digestive tract-derived neurohormonal dysregulation. This gut-centered model may provide insight into early, potentially reversible stages of the disease and identify a conceptual therapeutic target. Nonetheless, both the hypothesis and the accompanying surgical strategy—truncal vagotomy combined with proximal intestinal bypass—remain highly exploratory and require systematic validation through mechanistic and clinical studies. Further investigation is warranted to clarify the molecular regulation of I and S enteroendocrine cells, including the genetic and epigenetic factors that may drive hypersecretion. While speculative, interventions—surgical or pharmacologic—designed to modulate these digestive signals could represent a future avenue for research into T2DM prevention or remission, pending rigorous evidence.

## 1. Introduction

T2DM is a complex metabolic disease traditionally defined by insulin resistance, β-cell dysfunction, and chronic hyperglycemia. While these features have long been attributed to obesity, inflammation, and dysfunctional insulin signaling [1], increasing evidence over the past two decades has highlighted a central role of the gastrointestinal tract in T2DM pathogenesis. Observations from bariatric surgery, gut hormone modulation, bile acid signaling, and incretin-based therapies suggest that the gut actively contributes to the disease, beyond passive nutrient absorption [2,3,4].

Despite this growing body of evidence, a unifying pathophysiologic model that connects these diverse gut-related mechanisms is lacking. Although bile acid signaling, incretin secretion, and the effects of proximal intestinal exclusion have been individually studied, the findings remain fragmented. The absence of an integrated framework leaves a critical knowledge gap in our understanding of T2DM’s upstream origins.

This work builds upon the anti-incretin theory, which postulates that nutrient-stimulated neurohormonal signals—termed “anti-incretins”—arise in the proximal intestine to counterbalance incretin effects and maintain glycemic homeostasis. Excess anti-incretin signals, perhaps stimulated by macronutrient composition or chemical additives in modern diets, might cause insulin resistance, reduced insulin secretion, and β-cell depletion, leading to T2DΜ [2,5]. We hypothesize that the neuroendocrine signals produced by I and S cells in the proximal intestine (duodenum and proximal jejunum) function as endogenous anti-incretins.

In this context, we hypothesize a novel model centered on the chronic overstimulation of I and S cells by high-fat, high glycemic index modern diets. This drives what we term “amplified digestion”—a state marked by heightened vagal and hormonal stimulation of biliary and pancreatic secretions, increased enzymatic and bile acid activity, and alterations in bile acid composition. This condition leads to an extended breakdown of carbohydrates, lipids, and proteins into absorbable units, thereby promoting excessive nutrient absorption and ultimately contributing to insulin resistance and progressive β-cell failure.

To investigate this hypothesis, we synthesize clinical, surgical, and experimental findings into a cohesive, gut-centered framework that explains observed metabolic improvements following gastrointestinal interventions and informs future therapeutic strategies. Within this framework, we explore how targeted disruption of exaggerated digestive neurohormonal signaling—particularly via the combination of truncal vagotomy and gastric bypass—could potentially suppress overadaptive gut signaling and offer a novel, physiology-based approach to T2DM treatment.

Building on this, the hypothesis opens promising avenues for molecular-level research. Investigating genetic, epigenetic, or transcriptomic alterations in I and S secretory cells, along with their upstream regulatory stimuli, could reveal mechanisms underlying digestive hypersecretion and further support the reframing of T2DM as a neurohormonal disorder rooted in duodenal dysfunction.

## 2. Methods

This review is guided by the anti-incretin theory, introduced by Rubino in 2002, which posits that neurohormonal signals in the proximal intestine—termed “anti-incretins”—can become overstimulated by modern diets, contributing to insulin resistance and β-cell dysfunction [2,5].

Based on our extensive study of gastrointestinal physiology, we noted that the neuroendocrine cells most abundant in the proximal intestine—specifically, the I and S secretory cells—produce the dominant nutrient-stimulated neurohormonal signals in this region. We hypothesized that these mediators of digestion may act as endogenous “anti-incretins”. The question we aimed to answer was as follows: Could chronic overactivation of CCK and secretin pathways be a driving force behind insulin resistance and the development of T2DM? This reflection guided our investigation, leading us to examine the links between dietary patterns, digestive adaptation, and neurohormonal activity in the pathogenesis of metabolic dysfunction. The existing evidence allowed us to formulate a model in which overstimulation of digestive signals acts as a key driver of insulin resistance.

The anti-incretin theory also posits that under opposite conditions—namely, reduced nutrient stimulation via very low-calorie diets, accelerated intestinal transit (e.g., sleeve gastrectomy), or exclusion of the proximal intestine (e.g., gastric bypass)—anti-incretin signaling may decrease, thereby restoring metabolic balance. Based on this rationale, we conceptualized the antidiabetic mechanism of proximal intestinal exclusion as a reduction in nutrient-stimulated digestive neurohormonal signals.

The surgical strategy of combining truncal vagotomy with gastric bypass emerged as a natural conclusion of our research into gut physiology. To investigate its potential effectiveness, we reviewed retrospective studies of gastrointestinal surgeries performed for other indications—such as gastric cancer or peptic ulcer disease—in patients with T2D. These studies included various combinations of proximal intestinal bypass and truncal vagotomy, with or without gastrectomy, allowing us to assess their incidental antidiabetic effects.

## 3. Evidence Supporting the Hypothesis

### 3.1. Digestive Neurohormonal Regulation: From Physiology to Pathogenesis

**Figure 1:**
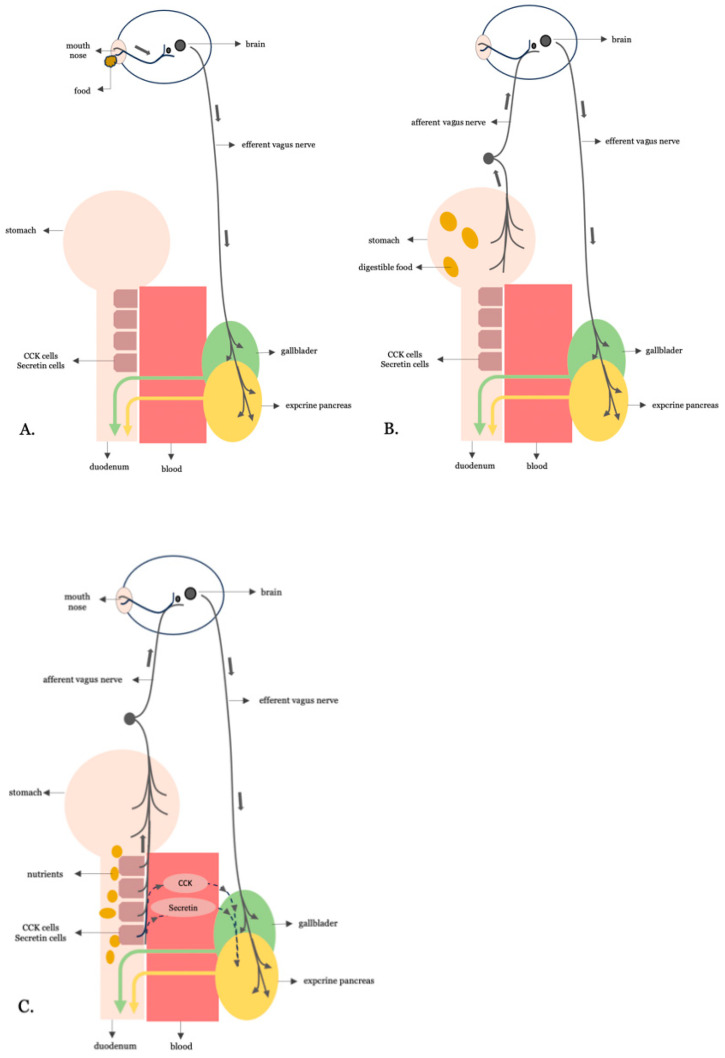
The three phases of neurohormonally regulated biliopancreatic secretion. (**A**) Cephalic phase: Anticipatory responses to sensory cues (e.g., taste, smell) trigger brain activation and efferent vagus nerve signaling to the gallbladder and pancreas. (**B**) Gastric phase: Gastric distension from ingested food activates vagal afferents, which elicit a vago-vagal reflex that enhances efferent vagal output to promote biliopancreatic secretion. (**C**) Intestinal phase: Nutrient-rich chyme entering the duodenum stimulates enteroendocrine I- and S-cells to release CCK and secretin, respectively.

Digestive activity is orchestrated through three interconnected neurohormonal phases of pancreato-biliary secretion: cephalic, gastric, and intestinal. These phases are initiated, respectively, by sensory stimuli, gastric distension, and chyme entry into the duodenum and are regulated by both vagal neural signals and enteroendocrine hormonal outputs [6,7] (Figure 1).

In the cephalic phase, sensory cues, such as the taste or smell of food, trigger brain activation and efferent vagal signaling to the gallbladder and pancreas. This anticipatory response primes the digestive tract by initiating modest enzyme and bile secretion even before any nutrient reaches the gut.

The gastric phase is triggered by gastric distension as food enters the stomach. Vagal afferents are activated and initiate a vago-vagal reflex that enhances parasympathetic output, leading to increased secretion of digestive enzymes and bile in anticipation of chyme reaching the intestine.

The intestinal phase, which is the most metabolically active and accounts for up to 70% of postprandial biliopancreatic secretion, begins when nutrient-rich chyme enters the duodenum. At this point, fats and proteins stimulate I and S secretory cells, while acids and fatty acids stimulate secretin-secreting S-cells. These enteroendocrine cells are densely located in the duodenum and proximal jejunum. The hormones they secrete activate both vagal reflexes and receptor-mediated signaling, leading to robust pancreatic enzyme secretion and gallbladder contraction, thereby amplifying digestive activity.

### 3.2. Is the Overstimulative Neurohormonal Digestive Axis the Missing Link Between Western Dietary Patterns and Insulin Resistance?

The link between the Western diet and metabolic disease is well established [8]. In humans, a high intake of processed carbohydrates, saturated fats, and low fiber is consistently associated with glucose dysregulation and an increased risk of T2DM [9,10]. Diets with a high glycemic load and elevated fat content induce exaggerated postprandial glucose and insulin responses, promoting the development of insulin resistance [11,12]. Interestingly, high-fat diets are commonly used to produce animal models that mimic human metabolic syndrome and T2DM, as they reliably induce insulin resistance, β-cell dysfunction, and obesity—closely recapitulating the human pathophysiology [13,14].

We suggest that such diets chronically overstimulate the neurohormonal digestive axis—particularly the pathways mediated by I and S secretory cells—leading to excessive nutrient absorption and ultimately contributing to insulin resistance.

Both animal and human studies have demonstrated that the activities of pancreatic digestive enzymes (e.g., amylase and lipase) and intestinal brush border enzymes (such as sucrase and isomaltase) are frequently elevated in T2DM. These elevations promote faster and more efficient hydrolysis and absorption of glucose and other nutrients. Large clinical studies, such as those derived from the LEADER trial, report that up to 23% of patients with T2DM have increased fasting amylase and lipase levels [15]. These enzyme elevations also correlate with blood glucose, insulin resistance, diabetes duration, and BMI [16,17,18]. Accelerated intestinal glucose absorption has also been documented in individuals and animal models with T2DM [19,20,21]. The state of excessive absorption is further amplified by increased bile acid activity and compositional changes, which enhance the micellar solubilization and uptake of dietary fats and carbohydrates and modulate gut hormone signaling that favors further nutrient absorption [22,23]. However, whether all these changes are drivers of hyperglycemia and metabolic dysfunction, secondary adaptations to the diabetic environment, or indicators of disease stage remains a subject that has yet to be clearly defined in the literature.

The therapeutic benefit of medications that inhibit these digestive processes—such as amylase/lipase inhibitors, alpha-glucosidase inhibitors (targeting brush border enzymes), and bile acid sequestrants—demonstrates that reducing enzymatic digestion and absorption improves glycemic control, slows diabetes progression, and can contribute to weight loss [24,25,26]. These findings suggest that digestive processes may play a contributory role in the development of insulin resistance and T2D, in part through hyperglycemia-driven molecular damage like AGE formation [27].

### 3.3. Validating the Anti-Incretin Hypothesis: Digestive Neurohormonal Hyperactivity

**Figure 2:**
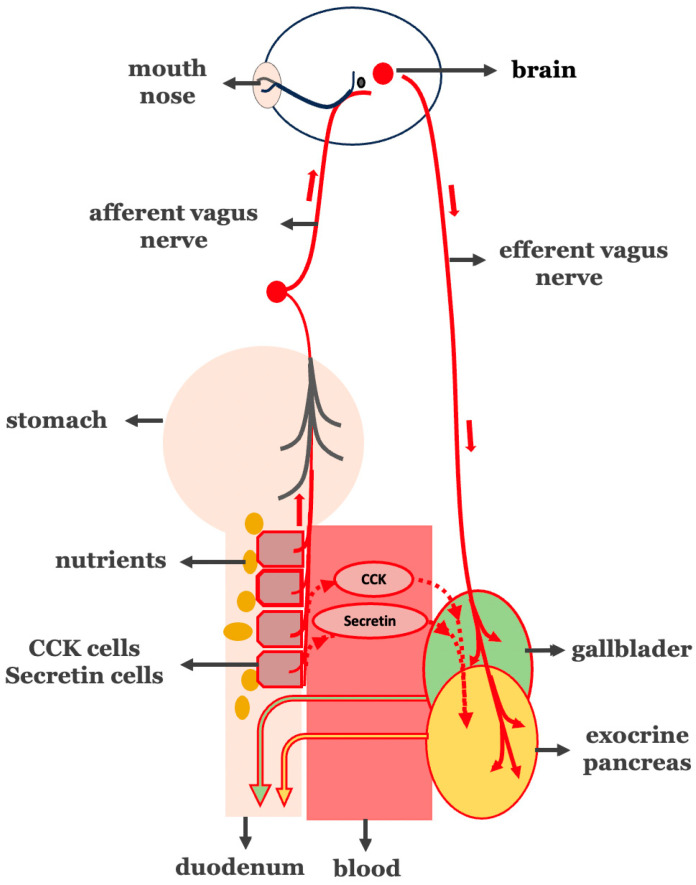
Pathological hyperactivation of the intestinal phase in biliopancreatic secretion. Overstimulation of I- and S-cells in the proximal intestine (circled in red) leads to increased secretion of both CCK and secretin (circled in red) and vago-vagal reflex-mediated (vagus nerve in red line) biliopancreatic secretion.

If the overstimulative neurohormonal digestive axis is a central driver of diet-induced insulin resistance, then—according to the anti-incretin theory—it may function as an endogenous “anti-incretin system”. In that case, the following should apply (Figure 2). A. As the vagus nerve serves as the neural component of the neurohormonal digestive axis, insulin resistance and T2DM should be associated with increased vagal nerve activity. B. The interruption of vagal signaling, such as through truncal vagotomy, should lead to improvements in insulin sensitivity and glycemic control. C. Since I and S secretory cells serve as the hormonal component of the neurohormonal digestive axis, insulin resistance and T2DM should be associated with excessive nutrient-stimulated hormonal signaling. D. Bypassing the duodenum—thereby minimizing nutrient-induced stimulation of neuroendocrine signals from I and S secretory cells—should contribute to the remission of insulin resistance and T2DM.

A thorough review of the literature provides evidence that indeed links neurohormonal hyperactivity and T2DM. The available impressive findings that support our hypothesis are presented below.

### 3.4. A Increased Vagal Activity in the Insulin Resistance Stage of T2DM

The association between obesity, hyperinsulinemia—as a hallmark of the insulin resistance stage in T2DM—and heightened parasympathetic nervous system activity has been recognized since the 1980s. Rohner-Jeanrenaud, a leading researcher in this field, demonstrated that in obese animal models, elevated insulin secretion is primarily mediated by increased vagal tone acting on pancreatic β-cells [28,29,30]. Subsequent studies have supported these findings, attributing the development and maintenance of hyperinsulinemia to an imbalance in autonomic nervous system activity—marked by increased parasympathetic and decreased sympathetic signaling within the gastrointestinal system [31,32].

### 3.5. B Truncal Vagotomy for an Improvement in Insulin Sensitivity in T2DM

The above-mentioned findings align with research studies that speculate truncal vagotomy—when performed as a stand-alone procedure for the treatment of morbid obesity—can normalize insulinemia and restore insulin sensitivity [28,33,34,35]. Moreover, truncal vagotomy has been consistently associated with reduced hunger, decreased food intake, and significant weight loss in individuals with severe obesity [36]. Although initially considered as a potential treatment for obesity in 1978, truncal vagotomy has not yet been systematically adopted for this purpose [37].

### 3.6. C Excessive Nutrient-Stimulated CCK and Secretin Hormonal Signaling in the Insulin Resistance Stage of T2DM

Physiological evidence strongly supports a link between nutrient-stimulated digestive hormones and the activity of I- and S-cells [38,39]. However, in the context of insulin resistance and type 2 diabetes mellitus (T2DM), the cellular and intracellular responses of these enteroendocrine cells to nutrient stimuli remain underexplored.

Transcriptional regulation of CCK and secretin secretion involves CCK-releasing factors (CCK-RFs), a group of peptide mediators that promote nutrient-responsive CCK secretion from duodenal I-cells—particularly following protein- or fat-containing meals [40,41]. Well-characterized factors include luminal CCK-releasing factor (LCRF), secreted by intestinal mucosal cells, which stimulates CCK release in a calcium-dependent manner, and monitor peptide (pancreatic secretory trypsin inhibitor I), released from the pancreas in response to luminal protein, which similarly enhances CCK secretion [39,42].

Although epigenetic regulation of these enteroendocrine pathways remains poorly defined, microRNAs have been identified in CCK-producing cells, suggesting possible regulatory roles [43]. Notably, although CCK gene expression remains unchanged in T2DM, patients show increased I-cell density in the large intestine and upregulation of CCK1 receptors in the duodenum [44]. These receptors, located on vagal afferent neurons, are key modulators of vagal tone and play a significant role in regulating digestive activity [45]. This observation may support the proposed theoretical model.

Animal models of insulin resistance—including high-fat-diet-fed and genetically obese mice—have been primarily used to investigate CCK expression in pancreatic islets, showing its upregulation and increased local CCK production during acute metabolic stress. However, there is a lack of evidence regarding CCK expression or activity in the duodenum in these models [46]. In humans with established T2DM, postprandial CCK responses are typically blunted rather than elevated [47,48]. This finding likely reflects a hallmark of advanced disease progression—consistent with the broader decline in enteroendocrine and exocrine pancreatic function observed in advanced T2DM [49]. 

### 3.7. D Proximal Intestinal Bypass Procedures Are Most Effective for the Remission of T2DM

The direct role of the proximal intestine (duodenum and proximal jejunum) in the development of T2DM was first demonstrated by Rubino et al. [50,51]. Their findings showed that excluding nutrient flow through the duodenum significantly improved glucose tolerance, while restoring duodenal passage in the same animals led to a recurrence of glucose intolerance.

Supporting this, several human studies have demonstrated that bariatric procedures involving bypass of the proximal intestine result in higher rates of diabetes remission compared to those that do not. In a cohort of 23,106 patients with metabolic syndrome, one-year remission rates for T2DM were significantly higher after gastric bypass (62%) and biliopancreatic diversion (74%) than after sleeve gastrectomy (52%) or gastric banding (28%) [52].

These findings have been corroborated by other researchers in both retrospective [53] and prospective studies [54] with follow-up periods of 2 and 3 years, respectively. Longer-term studies with follow-ups of 5 and 8 years further confirm the superiority of gastric bypass over sleeve gastrectomy—not only in achieving initial diabetes remission but also in maintaining it and reducing recurrence rates [55,56,57].

### 3.8. The Hypothetical Model for the Establishment of the Insulin Resistance Stage in T2DM

**Figure 3:**
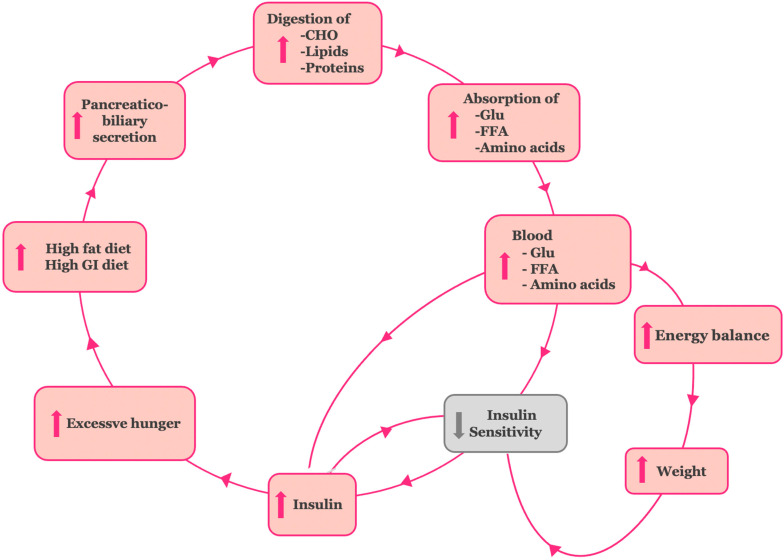
The hypothetical model for the establishment of the insulin resistance stage in type 2DM. Overstimulation of the neurohormonal digestive axis by high-fat, high glycemic index diets enhances biliary and enzymatic activity, increases nutrient absorption, and promotes hyperinsulinemia, excessive energy storage, weight gain, and increased hunger.

In Section 3.3 and Section 3.4, we substantiated the first four steps of the hypothetical insulin resistance model, demonstrating the transition from high-fat and high glycemic dietary intake to increased absorption of nutrient breakdown products (glucose, free fatty acids, and amino acids (Figure 3).

To construct a model consistent with the anti-incretin theory, we connected chronic exposure to amplified absorption of glucose, fatty acids, and amino acids with the onset of tissue insulin resistance, followed by β-cell dysfunction and, ultimately, T2DM. The evidence we gathered is as follows (Figure 3):

(a) Persistent elevation of blood glucose leads to a compensatory increase in insulin secretion by pancreatic β-cells in an effort to maintain normal plasma glucose levels. This sustained hyperinsulinemia plays a key role in the development of insulin resistance and is simultaneously reinforced by it [58]. The β-cells’ adaptation to this increased demand marks the early “adaptation stage” of T2DM.

(b) The excessive caloric intake associated with high-fat and high glycemic index diets, coupled with enhanced absorption of macronutrients, results in a positive energy balance and body weight gain. This process establishes obesity-related insulin resistance [59,60].

(c) The resultant hyperinsulinemia also increases appetite, promoting further overconsumption [61,62]. Together, these interactions form a self-perpetuating cycle, driving the insulin resistance stage of T2DM. At a later stage, though, pancreatic β-cell activity can no longer adequately meet the insulin demand created. The β-cell “hyper-stimulation” and subsequent “exhaustion” under gluco-, lipo-, and amino acid toxic conditions lead to pancreatic β-cell dysfunction and impaired insulin secretion. At the stage of β-cell dysfunction, hyperglycemia develops and T2DM is established [63].

### 3.9. Corresponding Corrective Antidiabetic Actions of Bariatric Surgery on the Hypothetical Model of Insulin Resistance

**Figure 4:**
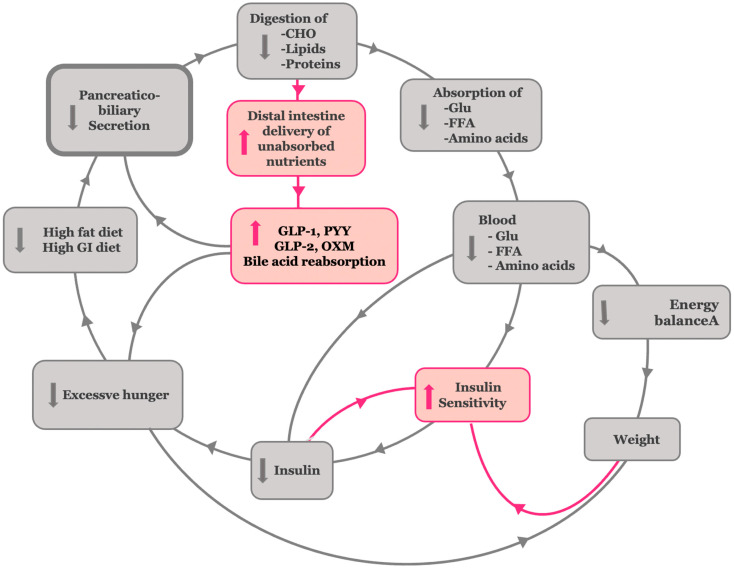
Corresponding corrective antidiabetic actions of bariatric surgery on the hypothetical insulin resistance model. Bariatric surgery reduces vagal and hormonal stimulation of biliopancreatic secretions, leading to decreased nutrient absorption. This, in turn, improves insulin sensitivity and mitigates excessive energy storage, weight gain, and hunger. Enhanced nutrient delivery to the distal gut boosts GLP-1, PYY, GLP-2, and OXD release and bile acid reabsorption, supporting β-cell function and glycemic control.

To align with the anti-incretin theory, bariatric procedures—particularly gastric bypass—are expected to exert antidiabetic effects by triggering processes that oppose those described in the insulin resistance model (Figure 4). A diminished anti-incretin signal reflects a weakened nutrient-stimulated neurohormonal digestive response, especially in the bypassed proximal intestine. This hypothesis is supported by the literature. Gastric bypass reduces early duodenal nutrient exposure, suppresses exocrine pancreatic secretion, and modifies both the timing and composition of bile acid delivery to the intestine [63,64,65,66]. Collectively, these physiological changes blunt—or even reverse—the overstimulated neurohormonal digestive signal.

**Figure 5:**
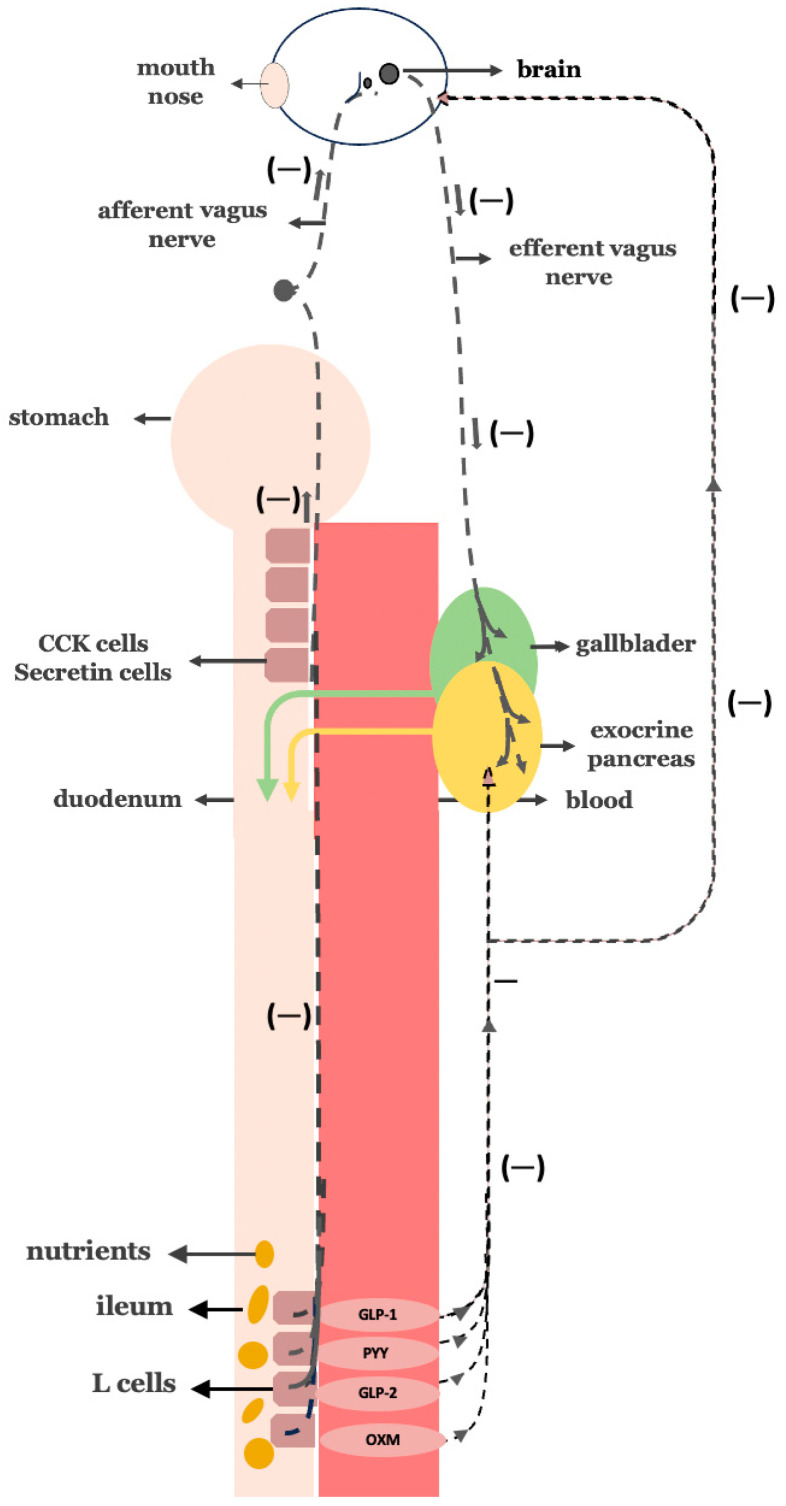
Negative feedback mechanism—The Ileal Brake: Once nutrients reach the distal intestine (ileum), L-cells are activated to release hormones such as GLP-1, PYY, GLP-2, and oxyntomodulin. These hormones signal the suppression of further pancreatic exocrine secretion and slow gastrointestinal motility, suppressing appetite but also boosting insulin secretion.

Continuing the hypothetical reasoning, the postoperative reduction in biliopancreatic secretions diminishes the enzymatic processing of carbohydrates, fats, and proteins in the proximal intestine, thereby reducing the absorption of glucose, free fatty acids, and amino acids [64,65]. This initial reduction in nutrient absorption helps blunt postprandial hyperglycemia and lowers circulating insulin levels, thereby improving insulin sensitivity [58]. The presence of undigested carbohydrates in the gut—acting as low glycemic index and load nutrients—together with reduced absorption of free fatty acids and amino acids, independently leads to an improvement in insulin sensitivity [66,67,68]. Moreover, the delivery of partially digested or undigested chyme and bile to the distal ileum results in supraphysiologic stimulation of L-cells, thereby activating the ileal brake (Figure 5). This brake is a key physiological feedback mechanism triggered by the presence of undigested nutrients—particularly long-chain fatty acids and complex carbohydrates—in the distal intestine. It stimulates the release of enteroendocrine hormones, such as glucagon-like peptide-1 (GLP-1), peptide YY (PYY), oxyntomodulin, and GLP-2 [6].

Following bariatric procedures—especially gastric bypass—the ileal brake becomes overactivated, markedly increasing the secretion of these hormones [69,70]. In turn, this hormonal surge not only suppresses appetite and promotes weight loss but also enhances β-cell function, boosts insulin secretion, and suppresses glucagon release, thereby contributing to improved glucose homeostasis [71,72,73,74,75].

Additionally, the exposure of the distal intestine to bile acids enhances their reabsorption and systemic circulation, which may explain the elevated bile acid levels observed after procedures such as Roux-en-Y gastric bypass and biliopancreatic diversion. These bile acids function as metabolic signaling molecules that promote glucose homeostasis, lipid metabolism, and energy expenditure. The shortening of the enterohepatic circuit in these surgeries facilitates earlier and more efficient bile acid uptake in the ileum, supporting their causal role in the metabolic benefits of bariatric surgery [4,76]. At the later stage of T2DM, the decrease in gluco-, lipo-, and amino acid-related toxicity reverts pancreatic islet β-cell dysfunction, leading to restoration of normal insulin secretion [63].

Beyond surgical modulation, pharmacological interventions targeting the neurohormonal axis have emerged as complementary strategies in the management of T2DM. GLP-1 receptor agonists effectively stimulate key components of the ileal brake mechanism. By activating GLP-1 receptors, these agents slow gastric emptying, enhance glucose-dependent insulin secretion, suppress glucagon release, and promote satiety—all effects that mirror the physiological outcomes of L-cell stimulation in the distal gut [77,78]. Their therapeutic efficacy in lowering blood glucose and promoting body weight loss underscores the role of distal gut-derived hormonal signaling in metabolic regulation. The success of GLP-1 analogs, therefore, supports the concept that restoring or mimicking the ileal brake constitutes a rational and effective strategy for disrupting the pathogenic cycle of insulin resistance and β-cell dysfunction that underlies T2DM.

### 3.10. Disrupting the Overactive Digestive Neurohormonal Axis: Truncal Vagotomy Combined with Gastric Bypass as a Targeted Strategy for Diabetes Remission

**Figure 6:**
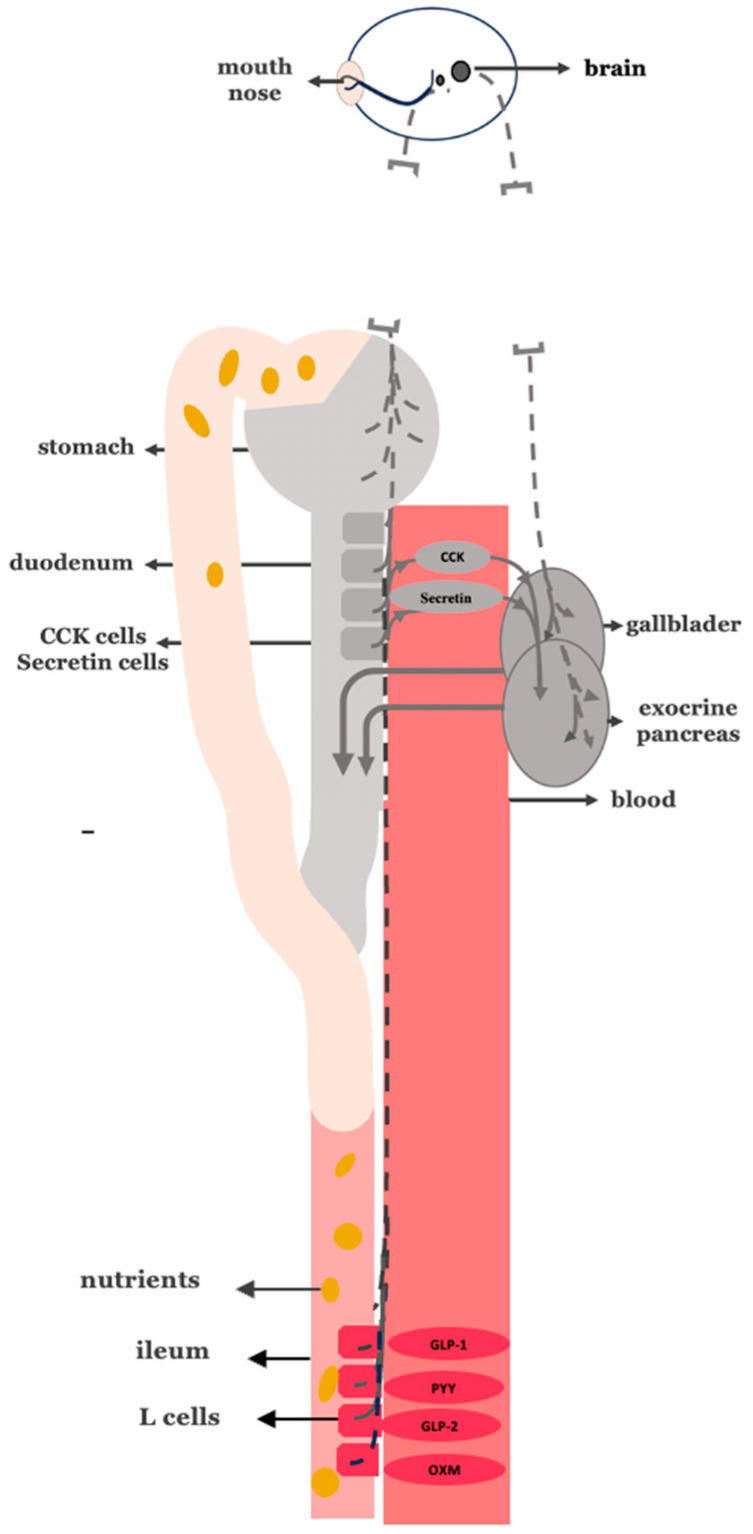
Truncal vagotomy in combination with gastric bypass. Gray stomach: no food passage. Gray proximal intestine: no nutrient passage. Gray enteroendocrine cells: reduced stimulation of CCK and secretin secretion. Vagal interruption: truncal vagotomy depicted as vagus nerve discontinuity. Gray gallbladder/pancreas: reduced biliopancreatic secretion. Red distal intestine: increased exposure to undigested nutrients, stimulating GLP-1, GLP-2, and PYY and oxyntomodulin release.

Building on the physiological insights gained from our investigation into the neurohormonal regulation of digestion, we conceptualized a surgical strategy aimed at interrupting the pathological overstimulation of biliopancreatic secretion observed in T2DM. This approach—combining truncal vagotomy with gastric bypass—emerged as a natural extension of our hypothesis (Figure 6). Rather than solely relying on nutrient rerouting, this dual intervention targets both vagal and hormonal drivers of digestive hyperactivity. The intended goal is to promote deeper and more durable glycemic improvements in individuals with T2DM [63].

According to our neurohormonal framework, gastric bypass alone reduces biliopancreatic output primarily by interrupting the gastric phase and part of the intestinal phase of digestive secretion. In contrast, truncal vagotomy uniquely suppresses all three phases of digestion—cephalic, gastric, and intestinal—by eliminating vagally mediated stimulation. Thus, combining truncal vagotomy with gastric bypass offers a synergistic, targeted antidiabetic mechanism of action that more comprehensively suppresses biliopancreatic secretion. This dual intervention may potentially yield superior metabolic outcomes in T2DM compared to gastric bypass alone.

Importantly, this combined approach may also provide greater long-term protection against body weight regain, a common limitation observed in patients undergoing standard bariatric procedures. By attenuating the cephalic phase of digestion, which contributes to early anticipatory hypersecretion, truncal vagotomy may reduce post-surgical drive for caloric compensation and limit the neurohormonal adaptations that promote weight recovery over time.

Longer disease duration and poor preoperative glycemic control are associated with lower remission rates and a higher risk of relapse. For this reason, we advocate for the early application of bariatric–metabolic surgery—particularly approaches incorporating truncal vagotomy—while islet β-cell function remains partially preserved and insulin resistance is still reversible. This strategy is also supported by our hypothetical neurohormonal model. Such early intervention may not only optimize initial metabolic outcomes but also enhance the long-term durability of glycemic control and weight loss.

### 3.11. Lessons from Upper GI Surgeries for Cancer and Peptic Ulcer Disease

Surgical procedures for gastric cancer and peptic ulcer disease in patients with T2DM—although primarily intended for oncologic or ulcer management—offer valuable insights into the potential antidiabetic effects of combining truncal vagotomy (TV) with gastric bypass techniques.

Several retrospective studies indicate that total gastrectomy, which includes TV, is associated with higher T2DM remission rates compared to subtotal or distal gastrectomy. Zhu et al. reported a 90.4% diabetes remission rate after total gastrectomy with Roux-en-Y reconstruction—the same configuration used in gastric bypass—compared to 65% and 29.3% for Billroth II and I, respectively, in non-obese patients (mean BMI 22.4 kg/m^2^) [79]. Wang et al. found a 75% remission rate with total gastrectomy versus 26.1% with subtotal gastrectomy, despite similar BMI and reconstruction methods [80]. Additional studies by Lee et al. and Kim et al., as well as a meta-analysis by Peng et al., support these findings [81,82,83].

While some studies, such as those by An et al. and Park et al. [84,85], did not identify gastrectomy type as a predictive factor for T2DM remission, the overall pattern points to the possibility that procedures involving TV—particularly when combined with Roux-en-Y reconstruction—exert stronger antidiabetic effects. We hypothesize that what differentiated total from partial gastrectomy in terms of improving T2DM was not only the extent of gastrectomy, but also the inclusion of truncal vagotomy, which was performed only in the former. These outcomes may reflect suppression of exaggerated biliopancreatic signaling, in line with our neurohormonal hypothesis.

### 3.12. Potential Drawbacks and Limitations of Adding Truncal Vagotomy in Gastric Bypass Procedures

Achieving complete truncal vagotomy is a technically demanding surgical procedure that requires meticulous exposure of the distal 5 cm of the esophagus, including the resection of extra-esophageal pre-aortic and hepatic branches. Due to the anatomical complexity and variability, incomplete nerve resection remains a significant challenge in the field and may compromise the intended metabolic benefits of truncal vagotomy [86]. One of the most serious intraoperative risks associated with truncal vagotomy is esophageal perforation during dissection, which, although exceedingly rare, can lead to life-threatening complications. Additionally, the incorporation of truncal vagotomy may prolong operative time, particularly in patients with pre-existing perioperative morbidity, increasing the overall surgical risk [87].

From a postoperative perspective, truncal vagotomy carries its own set of potential complications. A notable concern is post-vagotomy diarrhea, which is clinically significant in approximately 5% to 10% of patients and may affect quality of life [88]. Furthermore, when truncal vagotomy is combined with gastric bypass, it may exacerbate known complications of bypass procedures, such as steatorrhea, anemia, and osteoporosis, due to increased intestinal malabsorption of macronutrients, vitamins, and minerals [89]. Interestingly, the incidence of dumping syndrome appears to be lower following truncal vagotomy (20%) than gastric bypass alone (40%), indicating that vagal denervation may offer some protective effect against this syndrome [90].

## 4. Discussion

We hypothesize that a subset of T2DM may stem from chronic overstimulation of the neurohormonal digestive axis, marked by heightened bile acid, pancreatic, and intestinal enzyme activity—a condition that may lead to excessive nutrient absorption and, over time, drive insulin resistance and β-cell dysfunction. This hypothesis is supported by converging evidence from bariatric surgery outcomes, neurohormonal physiology, and preclinical models, which collectively lend plausibility to the hypothetical framework.

While our model centers on a duodenum-driven neurohormonal mechanism, it is not intended to supplant the multifactorial understanding of T2DM. Instead, we aim to complement established paradigms by proposing an upstream digestive contributor that may initiate or amplify known downstream pathophysiological processes. Key mechanisms such as (a) inflammatory adipokine signaling from visceral fat, (b) hepatic insulin resistance and ectopic lipid accumulation, (c) gut microbiota alterations and endotoxemia, (d) impaired glucose uptake in skeletal muscle, and (e) pancreatic β-cell dysfunction remain central to the disease. We suggest that chronic overstimulation of the proximal gut may interact with, or even trigger, these processes—particularly in early-stage or insulin-resistant phenotypes of T2DM. Thus, our model adds a proximal–gastrointestinal axis to the broader metabolic network implicated in diabetes, emphasizing its potential relevance in both pathogenesis and therapeutic innovation (Table 1) [91,92,93,94,95,96].

Despite the potential integrative value of this gastrointestinal model, several important limitations must be acknowledged. First, T2DM is a heterogeneous disease with diverse phenotypes, progression patterns, and responses to therapy. While our model may be applicable to insulin-resistant and reversal β-cell dysfunction forms of T2DM, it may not fully explain late-stage or insulinopenic diabetes.

Second, the possibility that bypassing the duodenum in combination with truncal vagotomy may interrupt the cycle of insulin resistance must be interpreted with caution. While data from upper gastrointestinal surgeries indicate possible antidiabetic effects, they are often retrospective and lack metabolic endpoints. The combination of truncal vagotomy with gastric bypass remains investigational, as no randomized clinical trials have yet evaluated its efficacy specifically in patients with T2DM. While theoretically sound, this strategy raises important clinical considerations, including technical complexity, nutritional sequelae, and long-term feasibility. The latter refers to the ability of the procedure to maintain durable metabolic benefits, minimize the risk of diabetes relapse, and remain safe and practical in the long run.

## 5. Conclusions

In this review, we hypothesize that overstimulation of neurohormonal digestive pathways may be an underappreciated contributor to the development of insulin resistance and T2DM. By highlighting the potential roles of the proximal intestine and vagally mediated digestive signaling, this hypothesis offers a novel lens through which to explore upstream metabolic dysfunction—distinct from conventional glucose-centric paradigms.

However, we recognize that these mechanisms remain hypothetical and require extensive experimental and clinical validation. The surgical strategy involving truncal vagotomy combined with proximal intestinal bypass represents a conceptually promising approach, though it remains investigational. It involves notable technical and ethical considerations and demands further scrutiny regarding safety, efficacy, and long-term metabolic outcomes.

Ultimately, our aim is not to supplant established models but to broaden the understanding of T2DM pathophysiology by incorporating gut-derived signals—particularly those relevant in the earlier, potentially reversible stages of the disease.

## 6. Future Directions

Future research should prioritize identifying genetic, epigenetic, or transcriptomic alterations in duodenal I- and S-cells—as well as aberrations in their upstream regulatory stimulation—that may predispose individuals to digestive hypersecretion and metabolic dysregulation. Understanding these mechanisms could uncover novel targets for intervention in the early pathogenesis of T2DM.

Novel therapies targeting the desensitization or modulation of these cells and/or their stimulation—potentially via surgical strategies like truncal vagotomy with proximal intestinal bypass or pharmacologic agents—may offer upstream intervention points for diabetes prevention and treatment. Prospective clinical trials should investigate whether early implementation of combination procedures that inhibit both neural and hormonal contributors to an over-stimulative digestive axis can induce durable remission of T2DM in individuals with reversible β-cell dysfunction. Ultimately, reframing T2DM as a gut-amplified disorder opens new avenues for prevention, personalization, and long-term metabolic control.

## Figures and Tables

**Table 1:** Core mechanisms of type 2 diabetes mellitus.

Target Organ(s)	Origin	Mechanism	Pathophysiological Features	Potential Interventions
Adipose tissue, [91]	Excess visceral adiposity	Inflammatory adipokine signaling from visceral fat	↑ TNF-α, IL-6, resistin; ↓ adiponectin → systemic inflammation → insulin resistance	Weight loss, TZDs, anti-inflammatory agents, lifestyle modification
Liver, [92]	High-fat diets, metabolic syndrome	Hepatic insulin resistance and ectopic lipid accumulation	↑ Gluconeogenesis; ↑ hepatic glucose output; lipotoxicity and hepatic steatosis	Metformin, GLP-1 RAs, low-carb diets, SGLT2 inhibitors, TZDs
Intestine, [93]	Western diet, antibiotics	Gut microbiota alterations and endotoxemia	Dysbiosis → ↑ LPS → metabolic endotoxemia → chronic low-grade inflammation	Prebiotics, probiotics, fiber-rich diet, GLP-1 Ras, metformin
Skeletal muscle, [94]	Physical inactivity, chronic nutrient overload	Impaired glucose uptake in skeletal muscle	↓ GLUT4 translocation; mitochondrial dysfunction; insulin signaling defects	Exercise, metformin, TZDs, weight loss
Pancreatic β-cells, [95]	Chronic glucolipotoxicity, genetic susceptibility, inflammation	Pancreatic β-cell dysfunction and failure	Progressive β-cell mass loss and impaired insulin secretion; oxidative stress, ER stress, islet inflammation	Early insulin, GLP-1 RAs, DPP-4 inhibitors, β-cell protective therapies
Pancreatic β-cells, [96]	Pancreatic β-cells with autoimmune destruction	Autoimmune-like features	Presence of GAD or other islet autoantibodies; β-cell destruction resembling T1DM	Early insulin therapy, immunomodulators (investigational)
Hypothetical: Duodenum	Hypothetical: Chronic high-fat, high glycemic index diets	Hypothetical: Amplified digestion–driven neurohormonal dysregulation	Hypothetical: Excessive vagal and hormonal (CCK, secretin) stimulation → increased biliopancreatic secretion → accelerated nutrient absorption → insulin resistance	Hypothetical: Truncal vagotomy+proximal intestinal bypass; vagal or hormonal pathway modulators (investigational)

Explanations: ↑ increase, ↓ decrease.

## Abbreviations

TNF-α	Tumor necrosis factor-alpha
IL-6	Interleukin-6
Ras	receptor agonists
TZDs	Thiazolidinediones
GLP-1	glucagon-like peptide-1
SGLT2	Sodium–glucose cotransporter 2
DPP-4	Dipeptidyl peptidase-4
LPS	Lipopolysaccharide
GAD	Glutamic acid decarboxylase
